# Atomistic Peptide Folding Simulations Reveal Interplay of Entropy and Long-Range Interactions in Folding Cooperativity

**DOI:** 10.1038/s41598-018-32028-7

**Published:** 2018-09-12

**Authors:** Jianlin Chen, Xiaorong Liu, Jianhan Chen

**Affiliations:** 1 Department of Hematology, The Central Hospital of Taizhou, Taizhou, Zhejiang 318000 P.R. China; 2 Department of Chemistry, University of Massachusetts Amherst, Amherst, MA 01003 USA; 3 Department of Biochemistry and Molecular Biology, University of Massachusetts Amherst, Amherst, MA 01003 USA

## Abstract

Understanding how proteins fold has remained a problem of great interest in biophysical research. Atomistic computer simulations using physics-based force fields can provide important insights on the interplay of different interactions and energetics and their roles in governing the folding thermodynamics and mechanism. In particular, generalized Born (GB)-based implicit solvent force fields can be optimized to provide an appropriate balance between solvation and intramolecular interactions and successfully recapitulate experimental conformational equilibria for a set of helical and β-hairpin peptides. Here, we further demonstrate that key thermodynamic properties and their temperature dependence obtained from replica exchange molecular dynamics simulations of these peptides are in quantitative agreement with experimental results. Useful lessons can be learned on how the interplay of entropy and sequentially long-range interactions governs the mechanism and cooperativity of folding. These results highlight the great potential of high-quality implicit solvent force fields for studying protein folding and large-scale conformational transitions.

## Introduction

The protein folding problem, i.e., understanding how natural proteins fold reliably into unique three-dimensional structures, remains one of the major tasks in molecular biology^1^. Significant advances in both theoretical and experimental aspects have been made in the last few decades and the general mechanism of protein folding is now considered to be known^2–5^. In particular, the statistical mechanics view, i.e., the energy landscape theory, has become a central framework for understanding protein folding, structure prediction and protein design^6–8^. The key ideas are that the global energy landscape of a naturally evolved protein resembles a partially rugged funnel and that the protein folds through multiple routes to the basin of global free energy minimum. The state-of-the-art explicit solvent protein force fields have also steadily improved over the years^9–13^, and achieved remarkable successes in recent atomistic protein folding simulations^5,14^. Nevertheless, a complete and unambiguous picture of protein folding at the atomic level has yet to emerge. For example, there are still controversies even on how a 16-residue β-hairpin from the C-terminus of the protein G B1 domain (GB1p) folds in isolation^15–25^. This reflects some of the limitations in current computational and experimental methodologies. Direct all-atom molecular dynamics (MD) or Monte Carlo (MC) simulations can provide insight at levels of detail and fast timescales that cannot be currently reached by experiments, but remain limited by accessible simulation timescales and force field accuracy. The speed limit of the fastest folding proteins ranges from a few microsecond (μs) to tens of μs^26^, mostly out of direct reach of statistically meaningful atomistic simulations when solvent is treated explicitly. As such, efficient implicit solvent models, where average properties of water are described macroscopically, can be particularly useful in reducing the timescale gap^27–32^.

An important advance in the experimental frontier of protein folding studies is the discovery and design of ultrafast folding peptides and proteins^33^. These systems are more amenable to detailed simulations and provide a unique opportunity to bridge the gap between theoretical and experimental investigations. In particular, empirical protein force fields, originally calibrated to characterize conformations near the native basin, have important limitations for protein folding studies^34,35^; virtually all of them tend to generate overly compact ensembles for unfolded protein states^36–38^. Availability of fast folding peptides makes it more feasible to recalibrate these force fields to include the non-native/unfolded conformational space^39^. Such optimization requires extensive conformational sampling and can substantially benefit from enhanced sampling methods, particularly the replica exchange (REX) class of sampling techniques^40–42^. For example, we have previous parameterized a generalized Born with smooth switching (GBSW)^43^ implicit solvent force field, by rebalancing solvation and intramolecular interactions to reproduce the experimental conformational equilibria of two helical peptides and a range of β-hairpins derived from GB1p^44,45^. These hairpins are sequentially homologous but display either reduced or enhanced stability compared to the native sequence^46^, offering excellent controls for optimization of various implicit and explicit solvent force fields^9,13,47,48^. The optimized GBSW force field demonstrates a good level of transferability, successfully folding both trpzip2^49^, a designed β-hairpin, and Trp-cage^50^, a designed mini-protein, to ~1.0 Å accuracy. It has also been successfully applied to calculate the conformational equilibria of small proteins^51–53^ and to describe non-trivial structural features of several IDPs^54–57^.

In the present study, we apply the optimized GBSW protein force field to examine the detailed thermodynamics and mechanisms of β-hairpin formation using the set of GB1p-derived peptides. Despite their small size, these hairpins are good model systems for understanding the folding of larger proteins, with cooperative folding transitions at multi-μs timescales and involvement of sequentially long-range hydrophobic and backbone hydrogen-bonding (H-bond) interactions^15^. The findings here are also made more relevant with the use of a single physics-based force field tuned to preserve the delicate balance of competing interactions. Coupling implicit solvent and REX enhanced sampling allows derivation of key thermodynamic properties with good statistical convergence for direct comparison with available experimental data on these peptides. Together, the results reveal an interesting interplay between chain entropy and long-range interactions in determining the cooperativity and mechanism of peptide folding.

## Results

### Structure and energetics of GB1p series β-hairpin folding

**Figure 1:**
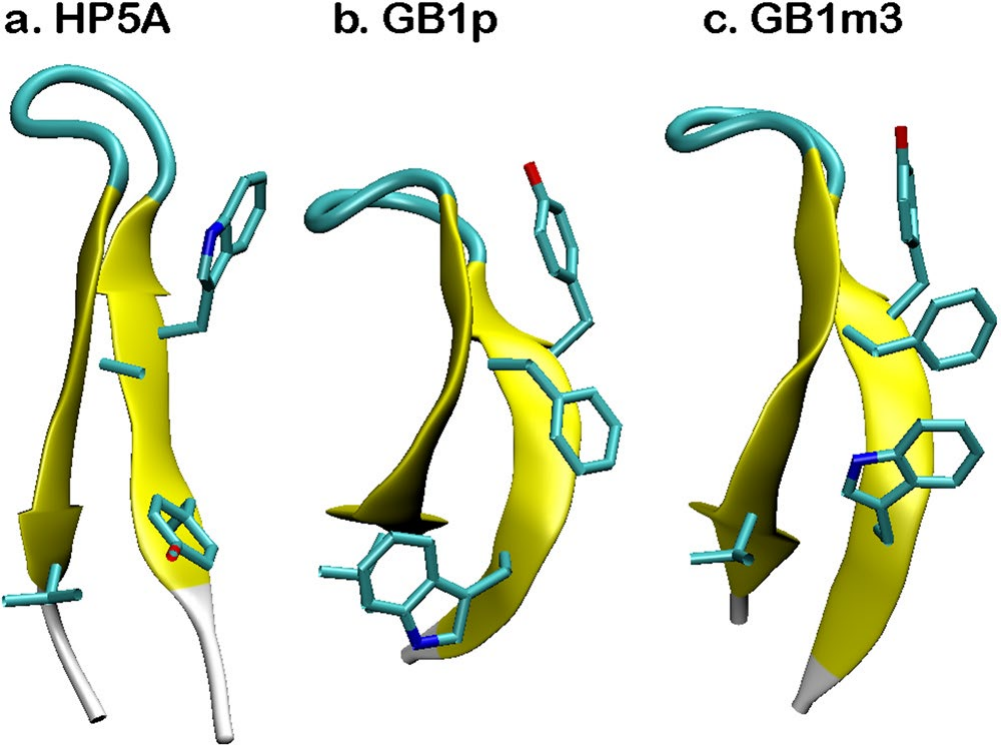
Representative folded structures of GB1p β-hairpins at 270 K. Key hydrophobic sidechains are shown in bonds. Peptide sequences and native contacts are given under Methods.

**Figure 2:**
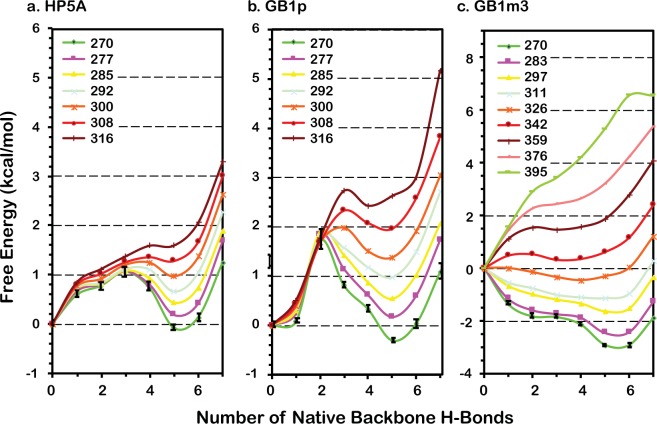
Free energy profiles of GB1p β-hairpins at different temperatures. All profiles were calculated directly from distributions sampled at various temperatures during the last 60 ns of REX-MD. Error bars are shown only for results at 270 K for clarity, which are standard errors estimated based on the differences calculated using first and second half of the production ensembles.

All three GB1p-series peptides can fold into β-hairpin structures in the GBSW implicit solvent (see Fig. 1), with the same backbone H-bond registry as observed in the intact protein G B1 domain. Key hydrophobic sidechains also form cross-strand contacts as expected. Interestingly, the hairpin structures formed by these peptides display significant differences in the level of curving, with HP5A showing the least bending. As it will be discussed later, this is likely due to different relative importance of hydrophobic side chain interactions and backbone H-bonds. The force field also correctly recapitulates the experimental observation that the folded hairpin state is the most stable for GB1m3 and least stable for HP5A^49^. Examination of the free energy profiles as a function of the number of native backbone H-bonds formed (*N*_HB_), shown in Fig. 2, suggests that all three hairpins fold cooperatively. It has been demonstrated that the number (or fraction) of native contacts formed provides an excellent reaction coordinate for describing protein folding using both coarse-grained^58^ and atomistic^26^ simulations. For both GB1p and HP5A, there are two well-defined folded and unfolded free energy minima, separated by a single major barrier. That is, both peptides display strong cooperative folding, with most native structural features formed together. This is highly consistent with previous experimental studies demonstrating that the isolated GB1p hairpin retains the folding cooperativity^15,18^. For GB1m3, there is a single free energy minimum that corresponds to the folded state at low temperatures, suggesting that it may follow ‘downhill folding’^59^. However, a mild free energy barrier is present near the melting temperature (*T*_m_), around 330 K (e.g., see the T = 342 K trace in Fig. 2c).

The cooperative folding behavior allows one to derive folding thermodynamics directly from the simulated ensembles sampled during REX-MD simulations (see Methods for details). The results for 270 K are summarized in Table 1 and compared with existing experimental data. Implicit solvent simulations were able to reproduce the order of stabilities of these hairpins, which is expected as they have been used in GBSW force field optimization^45^. Importantly, both folding enthalpy and folding entropy of GB1p derived from simulations are in quantitative agreement with values derived from NMR and T-jump tryptophan fluorescence experiments^15,18,46^. We note that implicit-solvent derived folding entropies do not include contributions from solvent. Nonetheless, the agreement re-affirms that the optimized GBSW force field offers a realistic balance between solvent-mediated protein-protein interactions vs. conformational flexibility, which may be directly attributed to the optimization strategy that involves both calibration of pair-wise interactions of backbone and side chain moieties and calculation of conformational equilibria of carefully selected model peptides^45^. Consistent with realistic estimation of folding entropy, the melting temperatures derived from REX-MD are also highly consistent with experimental results for all three hairpins^46^.

**Table 1:** Key folding thermodynamic parameters of β-hairpins at 270 K.

	Δ*G* (kcal/mol)		Δ*U* (kcal/mol)		Δ*S* (cal/mol/K)		*T*_m_ (K)	
	MD	Expr^a^	MD	Expr	MD	Expr	MD	Expr
HP5A	0.35 ± 0.1	0.82 ± 0.4	−2.4 ± 0.4		−7.6 ± 4	—	<270	<273^46^
GB1p	−0.44 ± 0.3	−0.8 ± 0.6	−9.9 ± 0.8	−11.6^15^	−38 ± 4	−39^15^	~273	<278^46^
				−12.6 ± 1.2^18^		−43 ± 4^18^		293 ± 4.3^18^
GB1m3	−1.3 ± 0.2	−1.1 ± 0.2	—	—	~−7.6	—	~330	333 ± 2^46^

Standard errors were estimated based on the differences calculated using first and second half of the production ensembles (last 60 ns of REX-MD).

^a^Estimated based on the folded population as Δ*G* = −RT ln *p*_f_/(1 − *p*_f_)^15,18,46^. Uncertainties are estimated using the range of reported *p*_f_.

**Figure 3:**
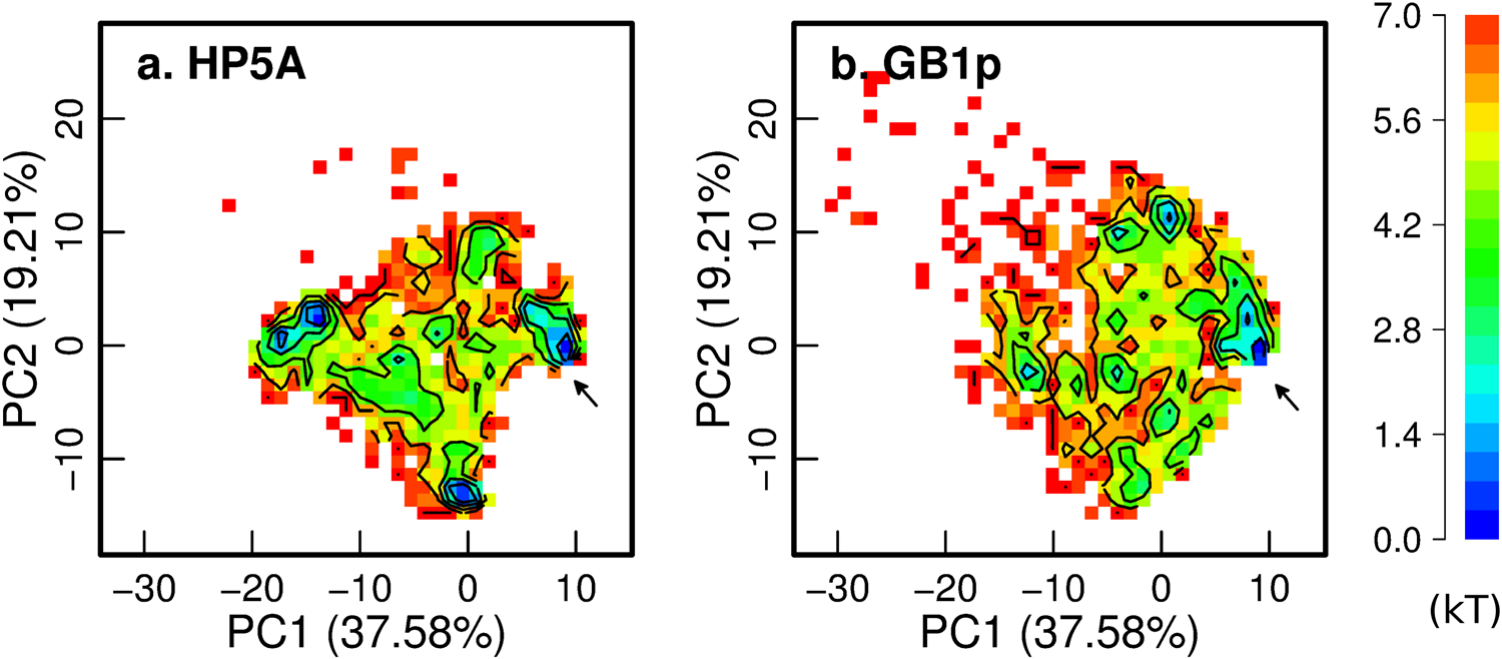
Free energy surfaces projected along the first two principal components. (**a**) HP5A and (**b**) GB1p. Only conformations sampled at 270 K during the last 60 ns of REX-MD were included and the principal components were derived by including backbone structures of both HP5A and GB1p. The arrows mark the location of folded basins. Contours are drawn at every kT.

**Figure 4:**
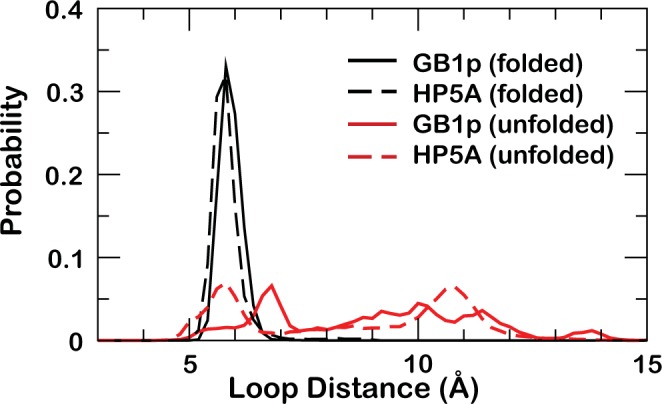
Distributions of loop distances in folded and unfolded GB1p and HP5A. The loop distance was calculated as the distance between Cα atoms of residues 6 and 11.

Even though both HP5A and GB1p hairpins are marginally stable (~35% vs ~65% folded), the underlying entropic and enthalpic contributions differ dramatically. The conformational entropic cost of folding for GB1p, ~38 cal/mol/K, is about 5-fold greater than for HP5A, which is only 7.6 cal/mol/K. This is mainly due to the proline-rigidified loop (-PATG-) in HP5A (and GB1m3), which was designed to replace the highly flexible -DATK- loop in the original GB1p sequence^46^. Analysis of the equilibrium conformational distributions, shown in Fig. 3, clearly shows that GB1p samples a wider conformational space compared to HP5A. In particular, HP5A samples two major nonnative basins beside the native state. Representative snapshots of these conformations are shown in Fig. S1, which suggests that the reduced conformational space sampled by HP5A can be primarily attributed to the loop. The loop forms a native-like turn in one of the nonnative states while adopting extended conformations in the other. This is also reflected in the loop distance distributions, where the -PATG- loop in HP5A occupied two major sub-states (dashed red line in Fig. 4) and has substantial probability of sampling folded like conformations (black traces in Fig. 4). In comparison, the -DATK- loop in GB1p rarely adopts folded-like configurations and can sample a broad and largely continuous range of disordered states (solid red trace in Fig. 4).

Intriguingly, the enthalpic stabilization of folding, −2.4 ± 0.4 kcal/mol, is also much smaller for HP5A, which is only about one fourth of that of GB1p (−9.9 ± 0.8 kcal/mol). The design of HP5A sequence involves replacement of larger hydrophobic contacts (Trp-Val and Tyr-Phe in GB1p and GB1m3) with the smaller ones (Trp-Ala and Tyr-Val), and at the same time introduces two Lys residues in the N-terminus to potentially stabilize the folded state via salt-bridge interactions with negatively charged C-terminus^46^. Yet, analysis of the contributions of various interactions, summarized in Table 2, revealed that the largest contribution to larger folding enthalpy of GB1p came from solvent-screened electrostatic contributions, which is ~7.0 kcal/mol stabilizing vs ~2.5 kcal/mol de-stabilizing for HP5A. Contributions of salt-bridge interactions to protein stabilities are highly context dependent, and it has been shown that exposed ones (such as those could potentially form between N-terminal Lys residues and C-terminal Glu and the carboxyl group) are often destabilizing or contribute only slightly to protein stability^55,60,61^. This seems to be true for HP5A as well. Instead, presence of larger sidechains in GB1p (and GB1m3) reduces the solvent exposure of backbone H-bonds and enhances their stabilities, leading to a much larger total electrostatic contribution to the folding enthalpy of GB1p. These energetic effects also likely explain higher bending of the folded hairpin structures observed for GB1p and GB1m3 (Fig. 1), which apparently increase the level of side chain-backbone contacts. We note that significant bending of GB1p hairpin structure was also observed in explicit solvent simulations^23^.

**Table 2:** Detailed folding energetics of HP5A and GB1p β-hairpins (all in kcal/mol).

	vdW	ASP	Elec	GB Ener	Elec + GB	Total
HP5A	−3.5 ± 0.1	−0.10 ± 0.1	12.2 ± 1.6	−9.7 ± 2.1	2.5 ± 0.4	−2.4 ± 0.4
GB1p	−0.50 ± 0.06	−0.40 ± 0.1	−14.9 ± 1.7	7.9 ± 2.1	−7.0 ± 0.4	−9.9 ± 0.8

Standard errors were estimated based on the differences calculated using first and second half of the production ensembles (last 60 ns of REX-MD).

### Cooperative folding of GB1p: hydrophobic interactions vs backbone H-bonds

**Figure 5:**
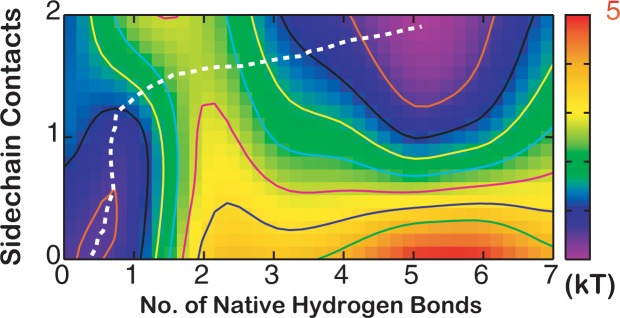
2D Free energy profiles of GB1p as a function of the numbers of native side chain contacts formed and backbone H-bond formed. The dashed line illustrates the minimal energy path connecting unfolded and folded states. Contours are drawn at every kT.

The ability of REX-MD simulations in GBSW implicit solvent to quantitatively recapitulate the stabilities and folding energetics of GB1p-series hairpins provides a unique opportunity to revisit several key questions regarding GB1p folding mechanism, particularly the roles of loop dynamics, hydrophobic side chain interactions and backbone H-bond formation. Folding of GB1p is clearly highly cooperative with a single major barrier separating the folded and unfolded states (Fig. 2), which is in agreement with existing experimental and computational studies^16,18,62,63^. Further examination of the 2D free energy surface demonstrates that the collapse of the peptide and formation of hydrophobic side chain contacts precede the backbone H-bond formation (e.g., see dashed line in Fig. 5). Such a sidechain driven cooperative folding mechanism is highly consistent previous NMR analyses^18,64^. A representative folding event sampled during REX-MD is shown in Fig. S2. Formation of native backbone H-bonds requires the loop making the correct turn and hydrophobic side chains making native-like contacts, which represent the rate limiting steps. Complete folding is almost always initiated by H-bonds near the turn, which leads to very fast zipping up of the rest of native backbone contacts (e.g., see Fig. S2). Consistent with simulations using latest optimized explicit solvent force fields^23^, there are no partially helical intermediate state not well populated at equilibrium (Fig. S3). Previous observation of such states was likely a consequence of inherent biases in early force fields, which have now been shown to yield over-stabilized helical and nonspecific compact states^9–13^. We also note that, despite the appearance of a single dominant folding pathway in Fig. 5, specific native side chain and backbone contacts can form in different orders, giving rise to relatively diverse microscopic folding pathways even for these small hairpins.

### Entropy, long-range interactions and folding cooperativity

**Figure 6:**
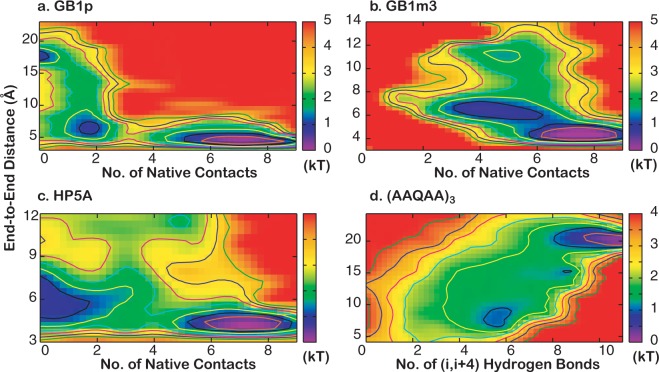
2D Free energy profiles as a function of the end-to-end distance and number of native side chain and backbone H-bond contacts formed. (**a**) GB1p, (**b**) GB1m3, (**c**) HP5A and (**d**) (AAQAA)_3_. Contours are drawn at every kT.

The funneled energy landscape theory predicts that cooperativity of folding arises naturally as a consequence of imperfect cancellation of (conformational) entropy and enthalpy^6–8^. The three GB1b-series hairpins simulated here provide a nice case study that illustrates how the magnitude of conformational entropy and strengths and (topological) distribution of native (sequentially) long-range interactions together determines the level of folding cooperativity. Folding of GB1p is the most cooperative among the three hairpins. Formation of multiple energetically favorable contacts is required in order to overcome the large conformational entropy of folding due to the flexible loop, which enforces cooperative folding. As shown in Fig. 6a, the peptide can readily sample collapsed conformations in the unfolded state where both sidechain hydrophobic contacts are frequently formed. However, it requires formation of about two additional backbone H-bonds to fully overcome the entropic cost and precede to the fully folded state sharply downhill in free energy (Fig. 2b). For HP5A, the entropic cost of folding is much smaller (Table 1), but the net energetic stabilization effects of side chain and backbone native contacts are also much smaller (Table 2). This leads a more gradual compensation of the entropic cost through formation of native contacts and folding does not turn downhill until over three backbone H-bonds are formed together with both side chain contacts (Figs 2a and 6c). In contrast, GB1m3 contains the same rigidified loop of HP5A but retains large hydrophobic side chains of GB1p. As a result, the modest entropic cost of folding can be readily compensated by formation of side chain hydrophobic interactions alone and the folding is virtually downhill as a function of the number of backbone H-bonds (Fig. 2c). Examination of the 2D free energy surface as a function of the end-to-end distance and number of native contacts also reveal minimal barriers (Fig. 6b). It resembles the surface for folding of helical peptide (AAQAA)_3_, where the entropic cost of helix-coil transitions is continuously compensated by formation of additional backbone H-bonds in a largely downhill fashion towards the folded state (Fig. 6d). However, there remains an important difference between folding of (AAQAA)_3_ helix and GB1m3 β-hairpin. While helix formation may be initiated virtually anywhere along the sequence of (AAQAA)_3_, the rigid loop of GB1m3 strongly restricts the accessible conformation space in the unfold state. Folding of the GB1m3 hairpin appears to largely follow similar folding pathways as observed in GB1p and HP5A, which is initiated near the turn and involve formation of hydrophobic side chain contacts followed by rapid zipping up of backbone H-bonds (e.g., see Fig. S2). Note that the coil state of (AAQAA)_3_ appears overly compact in GBSW compared to previous explicit solvent simulations^65^, and the actual folding landscape of (AAQAA)_3_ folding is thus likely more downhill than what is shown in Fig. 6d.

## Discussion

While the energy landscape theory has provided a general statistical mechanics framework for understanding the thermodynamics and kinetics of protein folding, atomistic simulations using physics-based empirical energy functions are usually required to provide quantitative description of the detailed folding mechanism and energetics for a specific protein or peptide. This has increasingly become a reality with recent development of carefully optimized implicit and explicit solvent force fields^9,10,13,47,48^ as well as development of advanced sampling methods and powerful hardware^4,66^. In this work, we combine an optimized implicit solvent force field and REX-MD enhanced sampling to analyze the folding of three related GB1p-series β-hairpins. The calculation was able to quantitatively recapitulate known mechanistic features as well as folding thermodynamics of these model systems. The results nicely illustrate how conformational entropy and long-range interactions together dictate various cooperative and downhill-like folding behaviors. This case study highlights the great potential of combining atomistic force fields and enhanced sampling for predictive studies of protein folding and large conformational transitions associated with various biological processes.

## Methods

### Model Peptides

Four peptides are used in this study, including helical (AAQAA)_3_ and three GB1p-series β-hairpins. Consistent with the experimental conditions^18,46,67^, termini of (AAQAA)_3_ peptide were blocked with Ace and NH2 respectively, and all hairpins with unblocked termini. The β-hairpin sequences are: G_41_EWTY DDATK TFTVT E_56_ (GB1p), KKYTW NPATG KATVQ E (HP5A), and KKWTY NPATG KFTVQ E (GB1m3) (the loop segments are underlined). Note that HP5A and GB1m3 are derived from the native sequence of the C-terminal hairpin (residues 41–56) of the B1 domain of protein G (GB1p) with modified stabilities (folded populations at 298 K estimated from NMR chemical shirts^46^ shown in parenthesis): HP5A (21 ± 10%) < GB1p (ca. 30%) < GB1m3 (86 ± 3%). Note that the folded population of GB1p has also been estimated to be ~42% at 278 K based on NMR^68^ and ~80% at 273 K based on tryptophan fluorescence experiment^15^. Native side chain contacts (for Gb1p) include: Trp_43_-Val_54_ and Tyr_45_-Phe_52_; a total of 7 native backbone contacts for these hairpins include: Glu_42_ NH-Thr_55_ CO, Glu_42_ CO-Thr_55_ NH, Thr_44_ NH-Thr_53_ CO, Thr_44_ CO-Thr_53_ NH, Asp_46_ NH-Thr_51_ CO, Asp_46_ CO-Thr_51_ NH, and Asp_47_ CO-Lys_50_ NH. (AAQAA)_3_ was estimated to be about 50% helical at 270 K based on NMR chemical shifts^67^.

### REX simulations in GBSW implicit solvent

As previously described^45^, all peptides were simulated using the previously optimized GBSW protein implicit solvent force field^45^, which was built on the CHARMM22/CMAP all-atom force field^69–71^. GBSW is generalized Born (GB)-class of implicit solvent that describes the solvent as a high dielectric continuum. GB offers a pair-wise, analytical approximation for calculating the electrostatic solvation free energy and is particularly suitable for molecular dynamics (MD) simulations^72^. GBSW in particular employs a van der Waals (vdW)-based surface with a smooth dielectric boundary, and the effective Born radii (the key quantities in the GB approximation) are calculated by a rapid volume integration scheme that includes a higher-order correction term to the Coulomb field approximation^73^. Default GBSW parameters were used along with 50 Lebedev angular integration points and 24 radial integration points up to 20 Å for each atom^45^. The nonpolar solvation energy was estimated from the solvent-exposed surface area with a phenomenological surface tension coefficient of 0.005 kcal/mol/Å^2^.

REX-MD simulations in GBSW were performed using the MMTSB Tool Set^74^ together with the CHARMM program^75,76^. REX involves multiple copies (replicas) of the peptide simulated at different temperatures independently and replicas attempt to exchange simulation temperatures periodically using Metropolis criteria that preserve the detailed balance. Replicas can travel up and down the temperature space during REX, which facilitates barrier crossing and reduces the probability of being trapped in states of local energy minima. 16 replicas were used in all simulations. The temperatures were distributed exponentially between 270 to 400 K for HP5A and GB1p and 270 to 550 K for (AAQAA)_3_ and GB1m3^45^. SHAKE was applied to fix the lengths of all bonds with hydrogen atoms and a time-step of 2 femtoseconds (fs) was used. Temperature exchanges were attempted every 2.0 picoseconds (ps) of MD between neighboring replicas. The total simulation lengths were 20 nanoseconds (ns) for (AAQAA)_3_ and 100 ns for the β-hairpins. In this work we only analyze ensembles derived from folding simulations that were initiated from fully extended structures. Previous comparison with results from control simulations initiated from folded structures suggested that the simulated ensembles were well converged within 100 ns^77^.

### Structural and energetic analysis

The post-analysis was done largely with CHARMM and the MMTSB Tool Set. The helicity was computed from the average 1–4 H-bond frequency, identified when the distance between the carbonyl oxygen of residue *i*, O_*i*_, and the amide hydrogen of residue *i* + 4, HN_*i*+4_, d(O_i_···HN_i+4_) ≤ 2.6 Å. Similar distance criteria were used to count the number of native backbone H-bonds in the β-hairpins. Sidechains are considered to be in contact if the shortest distance among heavy atoms is no greater than 4.2 Å. All distributions (or equivalently free energy profiles) were derived from conformations sampled during the last 60 ns of REX simulations. For principal component analysis (PCA), backbone structures of both GB1p and HP5A were first aligned using Cα atoms and then projected onto the first two principal components (PCs) to generate the 2D distributions/free energy profiles.

For folding energetic analysis, all conformations sampled during the last 60 ns of REX at 270 K were assigned to the folded state if *N*_HB_ ≥ 4 and unfolded states if *N*_HB_ ≤ 1. The free energy of folding was then calculated as Δ*G* = −*RT* ln (*P*_f_/*P*_u_). The folding energy, Δ*U*, was calculated as the difference between average potential energies of the folded and unfolded sub-ensembles. Given Δ*G* and Δ*U*, the folding entropy was estimated as Δ*S* = (Δ*U* − Δ*G*)/*T*. For GB1m3, the free energy profile (and equivalently the ensemble distribution) is dominated by the folded state (Fig. 2c) and the unfolded minimum is absent. Δ*G* was thus estimated by setting *P*_u_ = 1 − *P*_f_. The low occupancy of the unfolded state at 270 K also prevents reliable estimation of Δ*U* directly from average potential energies. GB1m3 and HP5A contains the same rigidified loop and Δ*S* for GB1m3 was thus estimated to be similar to that of HP5A in Table 1. The folding transition temperature (*T*_m_) was estimated as the temperature where *P*_f_ = *P*_u_.

## Electronic supplementary material

Supplementary Information

## Data Availability

The data that support the findings of this study are available from the corresponding author upon reasonable request.
