# Supplementary material for: Atomistic Peptide Folding Simulations Reveal Interplay of Entropy and Long-Range Interactions in Folding Cooperativity
Source: Sci Rep. 2018 Sep 12;8:13668. doi: 10.1038/s41598-018-32028-7 (PMC6135771; doi:10.1038/s41598-018-32028-7)
Supplement: Supplementary file 1 — Supplementary Information [file 41598_2018_32028_MOESM1_ESM.docx]

Supporting Information

**Atomistic Peptide Folding Simulations Reveal Interplay of Entropy and Long-Range Interactions in Folding Cooperativity**

#### Jianlin Chen^1^, Xiaorong Liu^2^ and Jianhan Chen^2,3^*

^1^Department of Hematology, The Central Hospital of Taizhou

Taizhou, Zhejiang, 318000, P. R. China

^2^Department of Chemistry and ^3^Department of Biochemistry and Molecular Biology

University of Massachusetts Amherst, Amherst, MA 01003, USA

*Corresponding Author: Phone: 413-545-3386; Email: [jianhanc@umass.edu](mailto:jianhanc@umass.edu)


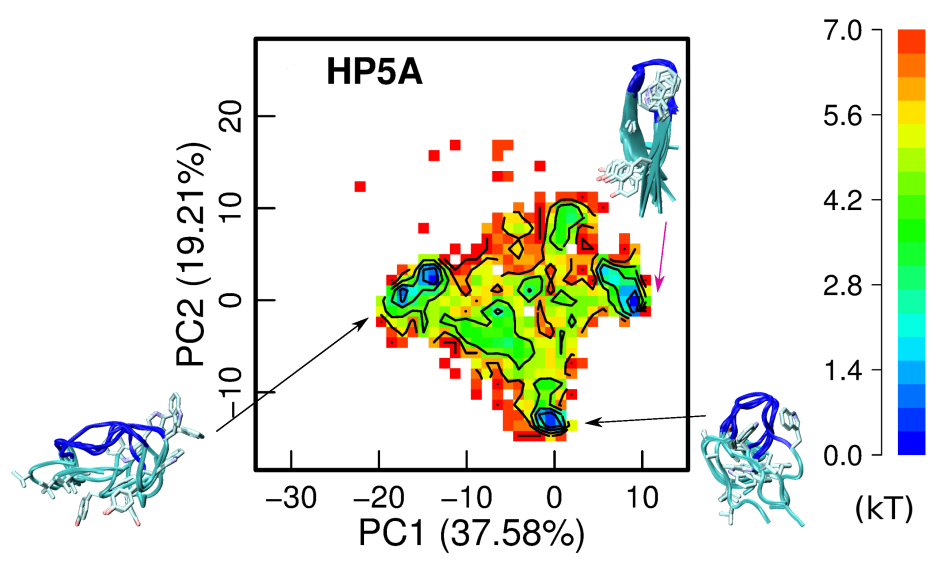


**Figure S1**. Representative folded and unfolded structures of HP5A. The loop region is shown in blue and hydrophobic side chains are shown in sticks.


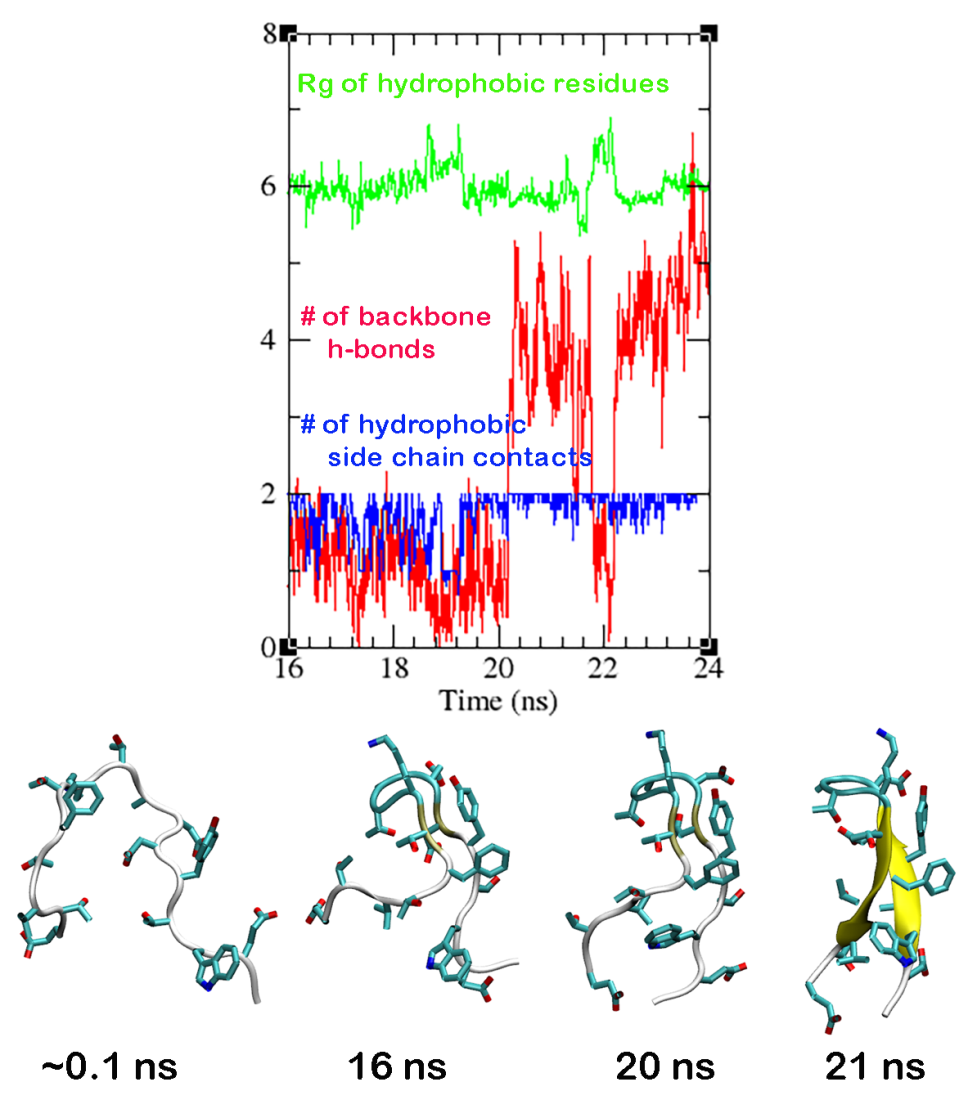


**Figure S2**. A representative folding event sampled during REX-MD simulation of GB1p hairpin. Similar folding pathways were observed for HP5A and GB1m3.


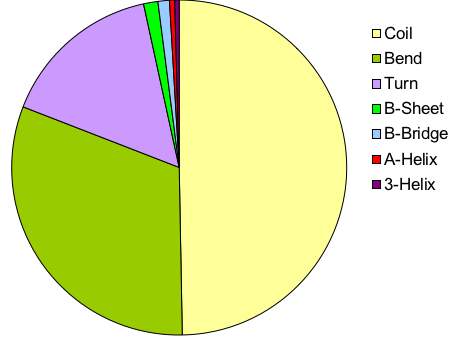


**Figure S3**. Distributions of secondary structures in the unfolded state of GB1p.
